# Bilateral Acute Angle-Closure Crisis Associated with Oral Tramadol Use After Robotic-Assisted Hysterectomy: A Case Report

**DOI:** 10.3390/reports9010024

**Published:** 2026-01-13

**Authors:** Assaf Kratz, Matan Bar, Ran Matlov Kormas

**Affiliations:** 1 Department of Ophthalmology, Soroka University Medical Center, Beer Sheva 8410101, Israel; 2 Faculty of Health Sciences, Ben-Gurion University of the Negev, Beer Sheva 8410501, Israel; 3 Division of Ophthalmology, Hadassah Medical Center, Faculty of Medicine, Hebrew University of Jerusalem, Jerusalem 9112001, Israel

**Keywords:** tramadol, angle-closure crisis, drug-induced, acute angle closure, case report

## Abstract

**Background and Clinical Significance**: Tramadol-associated acute angle-closure crisis is rare and has been reported only once previously following subcutaneous administration. Acute angle closure may occur in anatomically predisposed individuals in the setting of perioperative physiological stress, with medications acting as contributory factors. **Case Presentation**: A 38-year-old woman developed a bilateral acute angle-closure crisis shortly after initiating oral tramadol for postoperative pain relief following an uncomplicated robotic-assisted laparoscopic hysterectomy. Within 24 h, she experienced headache, nausea, vomiting, periocular pain, and blurred vision. Ophthalmic examination revealed markedly elevated intraocular pressure (45 mmHg OD, 39 mmHg OS), corneal epithelial edema, mid-dilated pupils, and completely closed angles on gonioscopy. Prompt intraocular pressure–lowering therapy followed by bilateral Nd:YAG laser peripheral iridotomy resulted in full anatomical and functional recovery, with visual acuity returning to baseline within 48 h. **Conclusions**: In this case, extreme anatomical susceptibility due to significant hyperopia and very short axial lengths likely played a dominant role, with perioperative physiological factors contributing to pupillary dilation. Oral tramadol may have acted as a permissive factor lowering the threshold for angle closure rather than as a sole causative agent. Awareness of this potential association is important to facilitate early ophthalmic referral and prevent unnecessary diagnostic evaluations.

## 1. Introduction and Clinical Significance

Acute angle-closure crisis is an ophthalmic emergency characterized by a sudden rise in intraocular pressure (IOP), leading to ocular pain, headache, nausea, corneal edema, and potential irreversible vision loss if not promptly treated. The condition typically occurs in anatomically predisposed eyes, with well-established risk factors including a shallow anterior chamber, short axial length, increased lens thickness, hyperopia, and a family history of angle closure [1,2,3]. These anatomical features are often bilateral, explaining the frequent involvement of both eyes during acute events.

Drug-induced angle closure represents an important secondary form of this condition and is commonly bilateral due to systemic medication exposure [4]. It may arise through pupillary block caused by mydriasis-inducing agents or through anterior rotation of the ciliary body and forward displacement of the iris–lens diaphragm, a mechanism associated with medications such as topiramate, phenothiazines, and sulfonamide derivatives [4,5,6,7,8,9,10]. Early recognition of drug-induced angle closure is clinically important, as discontinuation of the offending agent can significantly influence the clinical course.

Tramadol, an opioid analgesic structurally related to morphine and codeine, is widely prescribed for moderate-to-severe pain, particularly in postoperative settings [11,12]. Its pharmacologic effects include µ-opioid receptor agonism and inhibition of serotonin and norepinephrine reuptake, mechanisms that may lead to pupillary dilation through opioid or adrenergic pathways [13,14,15]. In anatomically predisposed individuals, such effects may lower the threshold for an acute angle-closure event.

Acute angle-closure glaucoma associated with tramadol appears to be rare and has been reported only once in the literature, exclusively following subcutaneous administration [16]. We report a case of bilateral acute angle-closure crisis following oral tramadol use in a hyperopic woman shortly after an uneventful robotic-assisted hysterectomy, highlighting a previously unreported route of exposure in this clinical context.

## 2. Case Presentation

A 38-year-old woman underwent an uncomplicated robotic-assisted total laparoscopic hysterectomy for adenomyosis. The surgical procedure and immediate postoperative period were uneventful, and she remained hemodynamically stable throughout. During the first 48 h after surgery, she received routine postoperative analgesia, including diclofenac 75 mg IM, dipyrone 1250 mg PO, and paracetamol 1000 mg IV, all of which provided adequate pain relief. On postoperative day 3, she met discharge criteria and was released home with oral Tramadol (Zaldiar 37.5/325 mg) prescribed for continued pain control.

Within 24 h of initiating Tramadol, the patient developed progressive and severe headache accompanied by nausea, vomiting, periocular pain, and blurred vision, symptoms that she initially interpreted as postoperative discomfort or dehydration. Due to the increasing intensity of her complaints, she sought medical evaluation and was assessed sequentially by an obstetrician-gynecologist, an internist, and a neurologist. Despite her significant symptoms, neurological examination remained unremarkable, and a CT scan of the brain demonstrated an empty sella but no acute intracranial abnormalities. No ocular etiology was initially suspected, and the patient continued to deteriorate symptomatically.

**Figure 1:**
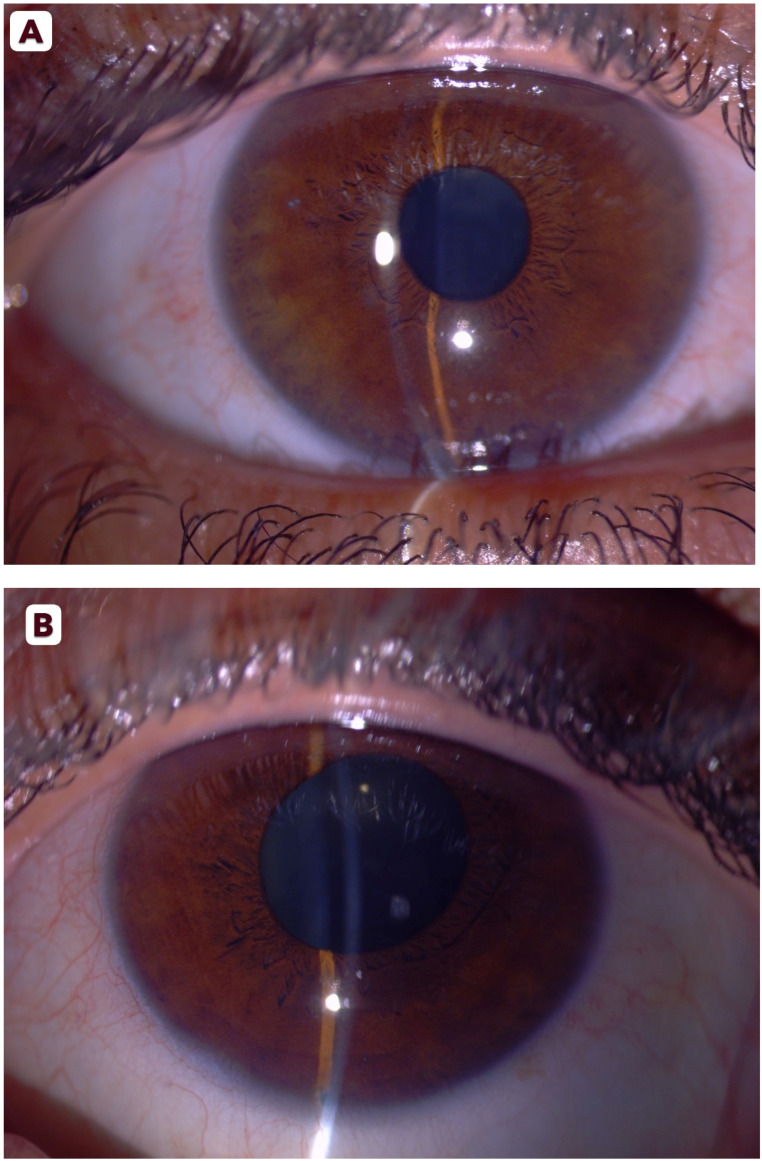
Slit-lamp photographs of the right (**A**) and left (**B**) eyes showing conjunctival injection, shallow anterior chambers, and mid-dilated pupils at presentation.

Upon ophthalmic referral, she reported a history of amblyopia in the left eye and bilateral hyperopia. Corrected visual acuity measured 20/40 OD and 20/60 OS. Slit-lamp examination revealed bilateral conjunctival injection, epithelial corneal edema, and shallow anterior chambers (Figure 1A,B). Pupils in both eyes were mid-dilated and sluggishly reactive to light. IOP measured 45 mmHg OD and 39 mmHg OS using Goldmann applanation tonometry. Gonioscopy demonstrated 360° appositional angle closure bilaterally without peripheral anterior synechiae. Bilateral fundus examination revealed healthy, pink optic disks with a C/D ratio of 0.4 OU, without retinal pathology or signs of glaucomatous optic neuropathy.

**Figure 2:**
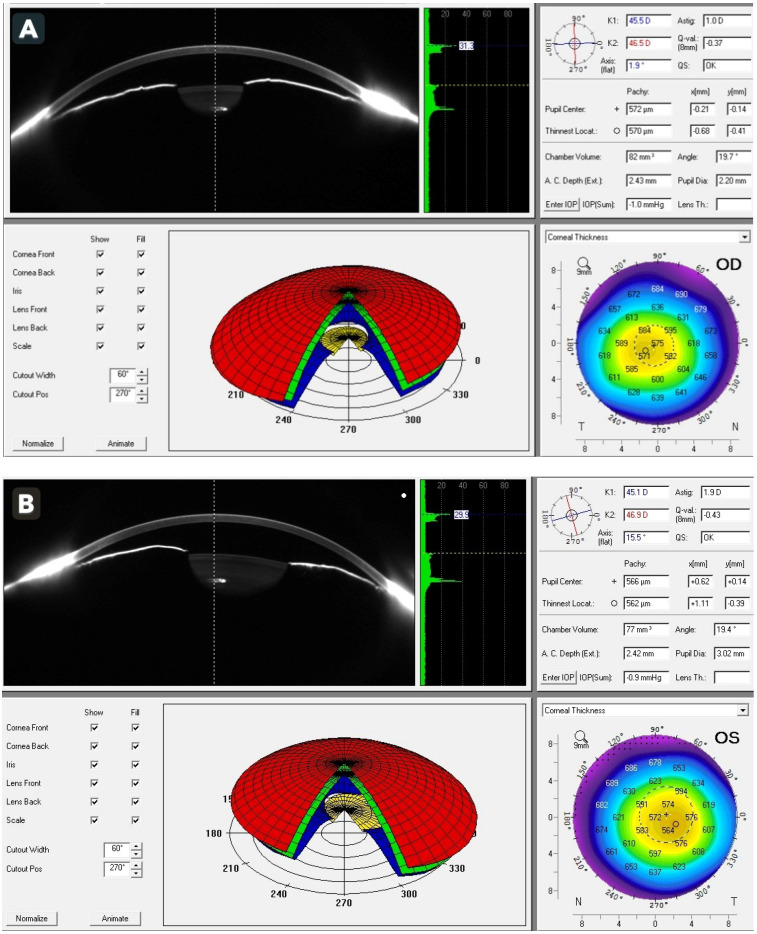
Scheimpflug imaging (Pentacam) of the right (**A**) and left (**B**) eyes demonstrating extremely shallow anterior chambers (82 mm^3^ and 77 mm^3^, respectively) and narrow chamber angles (19.7° and 19.4°, respectively).

Treatment was initiated immediately with intravenous acetazolamide together with intensive topical therapy, including dorzolamide 2%, timolol 0.5%, brimonidine 0.15%, latanoprost 0.005%, and pilocarpine 2%. Tramadol was discontinued at once due to the suspected drug-induced mechanism. The patient tolerated the treatment well, and over the subsequent several hours, IOP decreased significantly to 12 mmHg OD and 29 mmHg OS, accompanied by noticeable clinical improvement in corneal clarity and subjective relief of ocular discomfort. To further characterize the anterior chamber configuration after IOP reduction, additional anterior segment imaging was performed. Scheimpflug imaging (Pentacam, Oculus Optikgeräte GmbH, Wetzlar, Germany) demonstrated markedly shallow anterior chambers with chamber volumes of 82 mm^3^ OD and 77 mm^3^ OS and extremely narrow chamber angles measuring 19.7° and 19.4°, respectively. These findings supported the diagnosis of anatomical predisposition to angle closure and were consistent with hyperopic ocular morphology (Figure 2A,B). Auto-refraction further confirmed hyperopia, revealing +3.75 D OD and +4.75 D OS. Biometric assessment demonstrated short axial lengths, measuring 20.34 mm OD and 19.99 mm OS, reinforcing the presence of a structural susceptibility to angle-closure mechanisms and correlating well with the patient’s refractive and anterior segment characteristics.

Within 24 h of initiating medical therapy and discontinuing Tramadol, the patient became completely asymptomatic. Her headache, nausea, and periocular discomfort had resolved, and she reported a marked improvement in visual clarity. Visual acuity improved significantly, measuring 20/20 OD and 20/30 OS, and IOP stabilized at 10 mmHg bilaterally. With IOP adequately controlled and the corneas sufficiently clear to permit safe laser treatment, Nd:YAG laser peripheral iridotomies were performed in both eyes without complication. The procedures were well tolerated and resulted in immediate deepening of the anterior chamber periphery on clinical examination.

**Figure 3:**
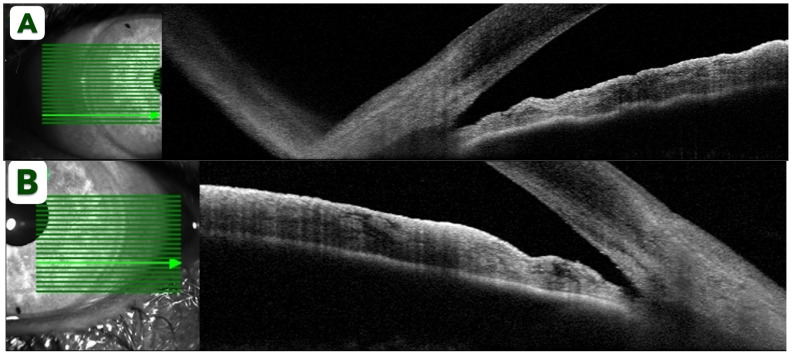
Spectralis AS-OCT of the right (**A**) and left (**B**) eyes after Nd:YAG iridotomy demonstrating partially opened narrow angles without peripheral anterior synechiae.

Post-procedure anterior segment imaging was obtained to evaluate the anatomical response. AS-OCT (Spectralis AS-OCT, Heidelberg Engineering GmbH, Heidelberg, Germany) demonstrated partially opened angles without evidence of peripheral anterior synechiae (Figure 3A,B). Although automated angle metrics are not provided by this device, manual measurements were performed to quantify angle opening. The mean angle opening distance at 500 µm from the scleral spur (AOD500) measured 0.20 mm in the right eye and 0.23 mm in the left eye, while the mean angle recess area at 500 µm (ARA500) was 0.09 mm^2^ and 0.10 mm^2^, respectively. These findings objectively confirm relief of the pupillary block component and restoration of aqueous outflow pathways [17]. Over the subsequent days, the patient continued to recover, and all topical medications were gradually tapered.

At one-month follow-up, IOP remained stable at 11 mmHg OU without the need for ongoing glaucoma therapy. OCT RNFL and standard automated perimetry were within normal limits, with no structural or functional evidence of glaucomatous damage, indicating complete resolution of the acute event and favorable mid-term outcomes. The patient expressed satisfaction with the outcome, and during subsequent follow-up no additional ocular complications or recurrent symptoms were observed.

## 3. Discussion

The acute angle-closure glaucoma crisis, often referred to as acute angle-closure glaucoma (AACG), is a sight-threatening ophthalmic emergency characterized by a rapid and substantial elevation of IOP due to abrupt obstruction of aqueous humor outflow. If not recognized and treated promptly, AACG may lead to irreversible optic nerve damage and permanent visual loss. The global burden of this condition is considerable, particularly in densely populated regions of Asia, where Primary Angle-Closure Glaucoma (PACG) is responsible for visual impairment and blindness at rates comparable to, or exceeding, those of primary open-angle glaucoma worldwide [1,2]. Importantly, a substantial proportion of AACG episodes have been reported as adverse reactions to systemic or topical medications, with some studies suggesting that up to one-third of acute angle-closure events may be drug-related. Recognition of the precipitating mechanism is therefore critical, as subsequent management strategies differ substantially according to the underlying pathophysiology [3,4].

Drug-induced AACG is broadly classified into two principal mechanisms: pupillary block and non-pupillary block. The pupillary block mechanism is the most common and typically occurs in eyes with pre-existing narrow iridocorneal angles. Systemic or topical agents with α_1_-adrenergic or anticholinergic properties may induce pupillary dilation, leading to obstruction of aqueous humor flow at the pupil, posterior pressure buildup, and forward displacement of the iris–lens diaphragm with secondary trabecular meshwork obstruction [2,3,4]. Numerous psychotropic agents, including tricyclic antidepressants, selective serotonin reuptake inhibitors, serotonin–norepinephrine reuptake inhibitors, and antipsychotics, have been implicated through this pathway, with serotonergic and noradrenergic activity contributing to passive or active mydriasis. In such cases, management consists of discontinuation of the offending medication and laser peripheral iridotomy, which is typically curative by equalizing pressure between the posterior and anterior chambers [2,3,4].

In contrast, the non-pupillary block mechanism represents an idiosyncratic drug reaction that may occur even in eyes without pre-existing narrow angles and is frequently bilateral. This mechanism involves ciliary body edema and choroidal effusion, resulting in anterior rotation of the ciliary body, forward displacement of the lens–iris diaphragm, and secondary narrowing of the anterior chamber angle. Sulfonamide-derived medications, particularly the anticonvulsant topiramate [5,6,7,8,9,10], are classically associated with this mechanism, which may also be accompanied by an acute myopic shift due to zonular relaxation. Several newer psychotropic agents, including venlafaxine, escitalopram, and bupropion, have also been linked to uveal effusion–related angle closure [5,6,7,8,9,10]. Because pupillary block is not the primary mechanism, laser peripheral iridotomy is ineffective in these cases, and management relies on immediate cessation of the causative drug, IOP reduction, and cycloplegia to deepen the anterior chamber [2,3,4,18,19,20,21].

Large population-based studies further emphasize the clinical relevance of drug-induced AACG. A recent analysis utilizing Korean national data identified 61 medications significantly associated with acute angle-closure events, with the highest odds ratios observed for centrally acting agents such as sumatriptan and duloxetine, in addition to topiramate [4]. Other implicated drugs included antidepressants, anxiolytics, antihistamines, and even non–central nervous system agents such as lactulose, with a median onset of AAC occurring within days to weeks after drug initiation. Demographic risk factors included older age and female sex, while anatomical predispositions such as short axial length, hyperopia, and shallow anterior chambers further increased susceptibility [3,4,18,19,20,21].

A summary of the major pharmacologic classes, representative agents, and proposed mechanisms associated with drug-induced acute angle closure is presented in Table 1, highlighting the diverse pathways through which medications may precipitate this potentially devastating condition.

Tramadol, an opioid analgesic widely prescribed for moderate-to-severe pain, is classically associated with miosis as part of its typical opioid effect [11,12]. However, paradoxical mydriasis has been reported in specific clinical settings, particularly in cases of overdose, altered metabolism, or hypoxic states, reflecting its complex pharmacologic profile that extends beyond pure opioid receptor agonism [13,14,15]. Tramadol metabolism involves multiple CYP450 pathways, and interindividual variations may potentiate adrenergic or serotonergic effects leading to mydriasis or forward rotation of the iris-lens diaphragm [11,12]. Such mechanisms mirror other drug-induced crises, including those associated with topiramate and sulfonamide derivatives

Tramadol exerts additional serotonergic and noradrenergic reuptake inhibition, which may influence autonomic control of the pupil and predispose susceptible individuals to pupillary dilation [12]. Mydriasis is a well-established precipitating factor for acute angle closure in anatomically predisposed eyes, as it promotes iris–lens apposition, pupillary block, and abrupt obstruction of aqueous humor outflow, resulting in a rapid rise in intraocular pressure [2,18,19,20,21]. Accordingly, the American Academy of Family Physicians emphasizes medication-induced pupillary dilation as one of the most common triggers of acute angle-closure glaucoma in susceptible patients, underscoring the clinical relevance of this mechanism [18].

Although tramadol was not identified among the 61 medications associated with acute angle closure in the population-based analysis [4], its central nervous system activity and combined serotonergic and noradrenergic effects place it within a broader pharmacological category that has previously been linked to angle-closure events.

To date, only a single case in the literature has described acute angle-closure glaucoma following Tramadol administration [16]. Mahmoud et al. reported a case of bilateral AACG occurring after subcutaneous Tramadol injections in a previously healthy 42-year-old man who presented with severe bilateral visual loss, headache, and vomiting several hours after drug administration. The clinical presentation was notable for markedly elevated intraocular pressure, mid-dilated pupils, corneal edema, and closed angles confirmed by gonioscopy and AS-OCT. The attack resolved following prompt medical treatment, discontinuation of Tramadol, and subsequent bilateral Nd:YAG laser peripheral iridotomy, with full visual recovery. The authors proposed that Tramadol may precipitate AACG through paradoxical mydriasis rather than its typical miotic opioid effect, mediated by adrenergic stimulation and inhibition of serotonin and noradrenaline reuptake [16].

In the present case, the patient’s markedly short axial lengths and significant hyperopia represent dominant predisposing factors for acute pupillary block, placing the eyes at the extreme end of anatomical susceptibility to angle closure. In such configurations, even minimal pupillary dilation may be sufficient to precipitate an acute event. The rapid onset of bilateral symptoms shortly after tramadol initiation suggests that the medication may have contributed to triggering the attack in this highly vulnerable setting. We acknowledge the absence of detailed dose–response information, including exact dosing frequency and cumulative exposure, and the limited ability to isolate its effect from overlapping perioperative factors. Postoperative pain, anxiety, sympathetic activation, and low-light environments, are well recognized causes of physiologic mydriasis and likely played a role in lowering the threshold for angle closure. Tramadol, through its central serotonergic and noradrenergic effects, may therefore be best viewed as a contributory or permissive factor rather than the sole causative agent. This case highlights that even medications not traditionally considered high risk may precipitate acute angle-closure crises in anatomically predisposed individuals, particularly when central nervous system–active agents are involved.

Importantly, the patient underwent multiple evaluations before ophthalmic referral, including neurological and systemic assessments, without recognition of the underlying ocular cause. Earlier consideration of an ophthalmic etiology in patients presenting with headache, nausea, vomiting, or blurred vision following recent medication changes may facilitate more timely referral to an eye-care professional and potentially prevent unnecessary investigations. Increased awareness of drug-induced angle-closure events across medical specialties may therefore improve diagnostic efficiency and patient outcomes.

## 4. Conclusions

This case highlights a rare but clinically significant adverse effect of Tramadol, demonstrating that oral administration may precipitate a bilateral acute angle-closure crisis in anatomically predisposed individuals. Although Tramadol is not traditionally regarded as a high-risk medication for angle closure, its central nervous system activity and serotonergic and noradrenergic properties may promote pupillary dilation and trigger a pupillary block mechanism in susceptible eyes. The rapid onset, bilateral presentation, and complete resolution following prompt discontinuation of the drug, medical therapy, and bilateral laser peripheral iridotomy underscore the importance of early recognition and timely ophthalmic intervention. Clinicians across medical specialties should consider acute angle closure in patients presenting with visual symptoms, headache, or nausea shortly after initiating Tramadol or similar centrally acting medications, particularly in those with known anatomical risk factors. Increased awareness of this uncommon association may facilitate earlier referral, prevent unnecessary diagnostic investigations, and reduce the risk of irreversible visual loss.

## Figures and Tables

**Table 1:** Main medications associated with drug-induced acute angle closure, grouped by pharmacologic class, proposed mechanism, and representative agents.

Drug/Class	Mechanism	Example Agents
Anticholinergics	Pupillary dilation (block)	Atropine, Tropicamide
Adrenergic agonists	Pupillary dilation (block)	Phenylephrine, Ephedrine
Antidepressants	Anticholinergic/serotonergic	TCAs, SSRIs, SNRIs, Duloxetine
Antipsychotics	Anticholinergic	Olanzapine, Quetiapine, Risperidone, Chlorpromazine
Sulfonamides	Ciliary body edema	Topiramate, Acetazolamide, Hydrochlorothiazide, Cotrimoxazole
Anticonvulsants	Uveal effusion	Topiramate, Zonisamide
Antihistamines	Mydriatic effect	Diphenhydramine, Chlorpheniramine, Loratadine, Cetirizine
Monoamine oxidase inhibitors	Mydriatic effect	Phenelzine, Tranylcypromine, Isocarboxazid
Metoclopramide	Unclear	Metoclopramide
Lactulose	Unclear	Lactulose

TCA: tricyclic antidepressants, SSRI: selective serotonin reuptake inhibitors, SNRI: serotonin–norepinephrine reuptake inhibitors.

## Data Availability

The data presented in this study are available on request from the corresponding author. The data are not publicly available due to patient confidentiality and institutional restrictions.

## References

[1] Foster P.J. (2002). The epidemiology of primary angle closure and associated glaucomatous optic neuropathy. Semin. Ophthalmol..

[2] Lowe R.F. (1970). Aetiology of the anatomical basis for primary angle-closure glaucoma: Biometric comparisons between normal eyes and eyes with primary angle-closure glaucoma. Br. J. Ophthalmol..

[3] Lachkar Y., Bouassida W. (2007). Drug-induced acute angle closure glaucoma. Curr. Opin. Ophthalmol..

[4] Na K.I., Park S.P. (2022). Association of drugs with acute angle closure. JAMA Ophthalmol..

[5] Senthil S., Garudadri C.S., Rao H.B.L., Maheshwari R. (2010). Bilateral simultaneous acute angle closure caused by sulphonamide derivatives: A case series. Indian J. Ophthalmol..

[6] Durai I., Dhavalikar M.M., Anand C.P., Ganesh V., Krishnadas R. (2016). Bilateral, simultaneous, acute angle-closure glaucoma in pseudophakia induced by chlorthalidone. Case Rep. Ophthalmol. Med..

[7] Chen S.H., Karanjia R., Chevrier R.L., Marshall D.H. (2014). Bilateral acute angle closure glaucoma associated with hydrochlorothiazide-induced hyponatremia. BMJ Case Rep..

[8] Achiron A., Aviv U., Mendel L., Burgansky-Eliash Z. (2015). Acute angle-closure glaucoma precipitated by olanzapine. Int. J. Geriatr. Psychiatry.

[9] Medeiros F.A., Zhang X.Y., Bernd A.S., Weinreb R.N. (2003). Angle-closure glaucoma associated with ciliary body detachment in patients using topiramate. Arch. Ophthalmol..

[10] Fraunfelder F.W., Fraunfelder F.T., Keates E.U. (2004). Topiramate-associated acute, bilateral, secondary angle-closure glaucoma. Ophthalmology.

[11] Tramadol Hydrochloride. FDA Drug Label. https://dailymed.nlm.nih.gov/dailymed/drugInfo.cfm?setid=dc420d50-6192-482d-b3da-3be55870a79a.

[12] Grond S., Sablotzki A. (2004). Clinical pharmacology of tramadol. Clin. Pharmacokinet..

[13] Tashakori A., Afshari R. (2010). Tramadol overdose as a cause of serotonin syndrome: A case series. Clin. Toxicol..

[14] Makris A., Matala M.E., Tsirigotis A., Karmaniolou I. (2012). Apnea and mydriasis after postoperative tramadol administration: An unusual complication and possible underlying mechanisms. Anaesthesia.

[15] Sansone R.A., Sansone L.A. (2009). Tramadol: Seizures, serotonin syndrome, and coadministered antidepressants. Psychiatry.

[16] Mahmoud A., Abid F., Ksiaa I., Zina S., Messaoud R., Khairallah M. (2018). Bilateral acute angle-closure glaucoma following tramadol subcutaneous administration. BMC Ophthalmol..

[17] Chen X., Wang X., Tang Y., Sun X., Chen Y. (2022). Optical coherence tomography analysis of anterior segment parameters before and after laser peripheral iridotomy in primary angle-closure suspects using CASIA2. BMC Ophthalmol..

[18] Michels T.C., Oana I. (2023). Glaucoma: Diagnosis and management. Am. Fam. Physician.

[19] Jain N.S., Ruan C.W., Dhanji S.R., Symes R.J. (2021). Psychotropic drug-induced glaucoma: A practical guide to diagnosis and management. CNS Drugs.

[20] Wu A.M., Stein J.D., Shah M. (2022). Potentially missed opportunities in prevention of acute angle-closure crisis. JAMA Ophthalmol..

[21] Gedde S.J., Chen P.P., Muir K.W., Vinod K., Lind J.T., Wright M.M., Li T., Mansberger S.L. (2021). Primary angle-closure disease preferred practice pattern. Ophthalmology.

